# A Simple, Quick, and Scalable Route to Fluorogenic Ubiquitin and Ubiquitin-Like Protein Substrates for Assessing Activities of Deubiquitinases and Ubiquitin-Like Protein-Specific Proteases

**DOI:** 10.1021/acschembio.5c00446

**Published:** 2025-08-13

**Authors:** Saibal Chanda, Alan Pham, Yifan Shi, Sandeep Atla, Wenshe Ray Liu

**Affiliations:** † Department of Biochemistry and Biophysics, College of Agriculture and Life Sciences, 14736 Texas A&M University, College Station, Texas 77843, United States; ‡ Texas A&M Drug Discovery Center and Department of Chemistry, College of Arts and Sciences, 14736 Texas A&M University, College Station, Texas 77843, United States; § Institute of Biosciences and Technology and Department of Translational Medical Sciences, School of Medicine, 14736 Texas A&M University, Houston, Texas 77030, United States; ∥ Department of Cell Biology and Genetics, School of Medicine, 14736 Texas A&M University, College Station, Texas 77843, United States; ⊥ Department of Pharmaceutical Sciences, Irma Lerma Rangel College of Pharmacy, 14736 Texas A&M University, College Station, Texas 77843, United States

## Abstract

Ubiquitin (Ub) and ubiquitin-like proteins (UBLs) regulate essential cellular processes as protein modifiers. While Ub signaling is well studied, many UBL pathways remain poorly defined, partly due to the limited availability of suitable UBL substrates. Here, we report the synthesis of fluorogenic Ub-ACA and UBL-ACA probes using the activated cysteine-based protein ligation (ACPL) technique to conjugate recombinant Ub and UBLs containing a *C*-terminal Gly to Cys mutation with glycyl-2-(7-amino-2-oxo-2*H*-chromen-4-yl)­acetic acid (Gly-ACA), a water-soluble fluorophore. This one-step strategy that allows replacing Cys with Gly-ACA enables simple, quick, and scalable synthesis of Ub-ACA and 11 UBL-ACAs. Five UBL-ACAs represent the first reported fluorogenic substrates for their respective UBLs. Afforded Ub-ACA and 10 UBL-ACAs were demonstrated to be active toward a panel of DUBs or UBL-specific proteases. Notably, SUMO4-ACA was cleaved by SENP1 with efficiency comparable to the other three SUMO-ACA probes despite SUMO4’s distinct structure compared to the other three SUMOs. In human cell lysates, all 12 probes are efficiently cleaved. URM1 has no known proteases. Our results indicate that URM1-specific protease(s) exist in human cells and are yet to be identified. Given their simple and scalable synthesis, these new fluorogenic Ub-ACA and UBL-ACA substrates are highly versatile tools for studying Ub and UBL pathways and drug discovery research.

## Graphical abstract

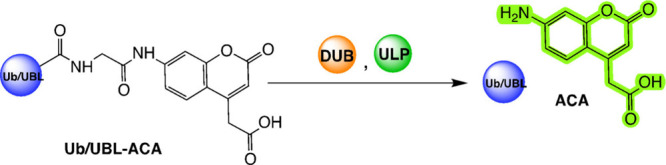

Ubiquitin (Ub) and ubiquitin-like proteins (UBLs) are small regulatory proteins that play essential roles in the post-translational modification of cellular proteins.^1^ Ub is best known for its role in targeting proteins for degradation via the Ub-proteasome system.^2,3^ Through an ATP-dependent enzymatic cascade involving an E1 activating, E2 conjugating, and E3 ligating enzyme, Ub is covalently attached to lysine residues on substrate proteins. This process is reversed by deubiquitinases (DUBs). Ub-involved pathways have been widely studied. Compared to Ub, the functions of UBLs are relatively less defined. Identified UBLs in human cells include SUMO isoforms (SUMO1–4), NEDD8, ISG15, FAT10, UFM1, URM1, MNSFβ, ATG12, GABARAP, GABARAPL1, GABARPL2, MAP1LC3A, MAP1LC3B, MAP1LC3C, and several others with no confirmed roles in protein modification.^4,5^ Most UBLs are conjugated to target proteins through dedicated E1-E2-E3 enzymatic cascades and removed by UBL-specific proteases (ULPs).^6^ URM1 is an exception. It is known to form a *C*-terminal thiocarboxylate, a process catalyzed by an E1-like protein in yeast and human cells.^7–9^ So far, there is no E2, E3, or ULP reported for URM1. For most other UBLs, their identified ULPs are either one enzyme or a group of enzyme homologues with several closely related members. Compared to Ub, which has more than 100 DUBs identified, all UBLs have a very narrow scope of ULPs reported. This is likely due to less research focused on them compared to Ub.

Ub-AMC, wherein 7-amino-4-methylcoumarin (AMC) is conjugated to the *C*-terminus of Ub, is the most widely used fluorogenic substrate for DUB research and is commercially available.^10,11^ Analogous UBL-AMCs, which are either commercially available or have been synthesized, include SUMO1-AMC, SUMO2-AMC, SUMO3-AMC, NEDD8-AMC, ISG15-AMC, and UFM1-AMC.^12–15^ Both Ub-AMC and UBL-AMCs are typically synthesized via expressed protein ligation (EPL). In this method, a recombinantly expressed Ub^1–75^- or UBL^–*c*Gly^-intein fusion (-*c*Gly: *C*-terminal glycine deletion) forms a thioester for a series of reaction treatments and finally with Gly-AMC to yield the desired product.^16^ However, this approach presents challenges, including difficulties in expressing and purifying labile Ub^1–75^- or UBL^–*c*Gly^-intein fusion proteins and low aqueous solubility of Gly-AMC, which can lead to reduced final yields. Despite the significance of DUBs as drug targets, the application of Ub-AMC in high-throughput screening (HTS) has been limited, partly due to constraints in substrate availability and cost. To address these limitations, we previously developed the Activated Cysteine-based Protein Ligation (ACPL) technique.^17,18^ This technique employs 2-nitro-5-thiocyanobenzoic acid (NTCB) to activate a cysteine residue in a protein for a one-step exchange reaction with an amine-containing compound to form a desired conjugation product (Figure 1A). Applying ACPL, we successfully synthesized Ub-AMC with improved reproducibility. Recognizing the solubility issues associated with Gly-AMC, we introduced Gly-ACA (glycyl-2-(7-amino-2-oxo-2*H*-chromen-4-yl)­acetic acid), a more water-soluble alternative. Utilizing Gly-ACA in the ACPL process enabled straightforward, one-step synthesis of Ub-ACA, further enhancing the practicality of the approach.^19^ In this study, we extend this approach to synthesize a series of UBL-ACAs as fluorogenic substrates for UBL-specific proteases. These substrates were employed to assess protease activities for both purified enzymes and in human cell lysates. Notably, for several UBLs, this represents the first report of corresponding fluorogenic probes. Our findings also suggest the presence of URM1-specific proteases in human cells, warranting further investigation. Additionally, we observed that SUMO4 serves as an efficient substrate for SUMO-specific proteases, comparable to SUMO1–3, despite its distinct structural features.^20^ Given that much is unknown surrounding many UBL-related pathways, these readily accessible fluorogenic probes, synthesized via the ACPL method using Gly-ACA, offer valuable tools for advancing biochemical and cell biological research in this domain.

**Figure 1:**
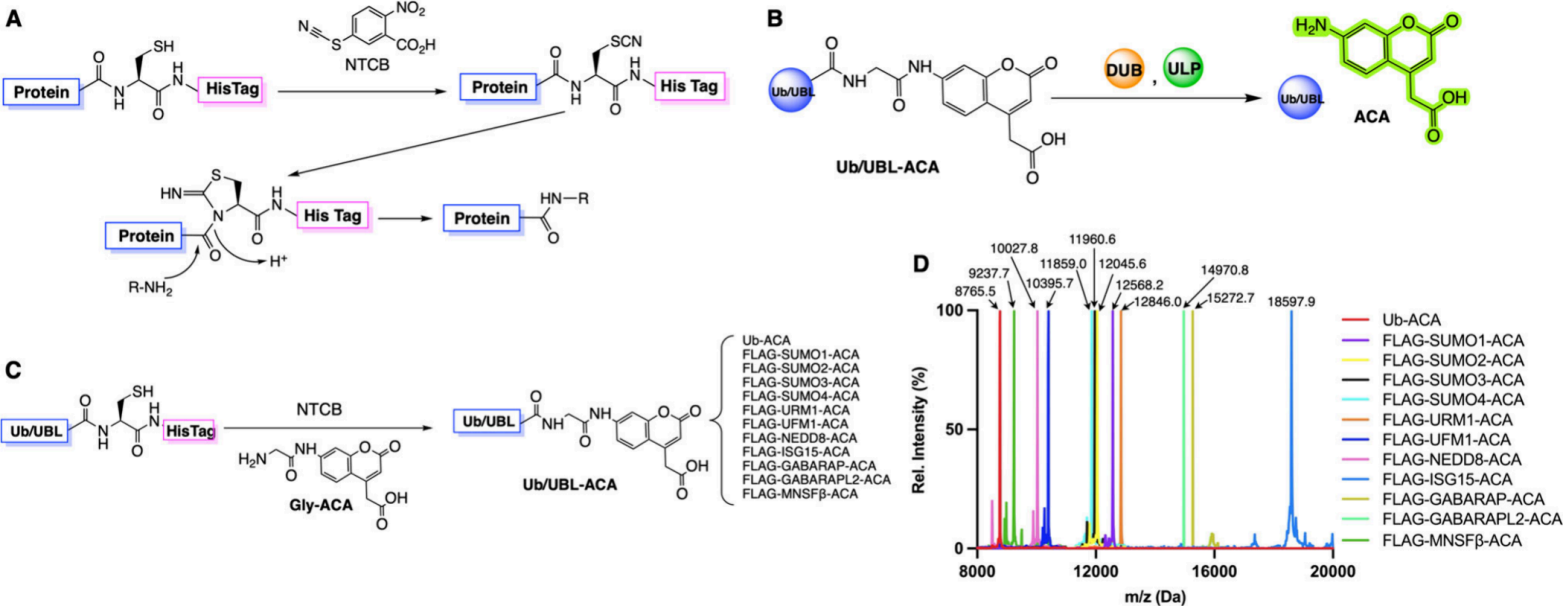
**Activated cysteine-based protein ligation (ACPL) technique and its use in the synthesis of fluorogenic Ub/UBL-ACA probes.** (**A**) Chemical mechanism of ACPL. A protein cysteine is activated by a nitrile donor molecule, e.g., 2-nitro-5-thiocyanatobenzoic acid (NTCB) to form a 1-acyl-2-iminothiazolidine intermediate that undergoes nucleophilic acyl substitution with an amine to generate a *C*-terminally functionalized protein. (**B**) Schematic illustration of Ub/UBL-ACA as a fluorogenic substrate for deubiquitinases (DUBs) and UBL-specific proteases (ULPs). Enzymatic cleavage at the Ub/UBL *C*-terminus releases the ACA fluorophore, resulting in a measurable fluorescence signal. (**C**) Summary of Ub-ACA and Flag-tagged UBL-ACAs used in the study and their synthesis. Each recombinant protein contains a Gly-to-Cys mutation at the terminal glycine position and a *C*-terminal 6×His tag. Recombinantly expressed and purified protein variants were based on Ub, SUMO1–4, URM1, UFM1, NEDD8, ISG15, GABARAP, GABARAPL2, and MNSFβ. All these proteins underwent a nucleophilic substitution with Gly-ACA in the presence of NTCB, leading to the formation of desired Ub/UBL-ACA probes. (**D**) Deconvoluted electrospray ionization mass spectrometry (ESI-MS) spectra of the final Ub/UBL-ACA probes. All spectra confirmed successful ligation with Gly-ACA, with observed molecular weights closely matching theoretical values (Δ ≤ 0.3 Da), verifying the identity and purity of the synthesized probes.

The ACPL technique employs a nitrile donor, such as NTCB, to activate a protein cysteine for its replacement with a small molecule amine. Using this strategy, we previously reported the synthesis of Ub-ACA by coupling Gly-ACA with recombinant Ub_1–75_-G76C-6×His. Here, we extended it to generate 11 UBL-ACAs. These UBL-ACAs are expected to serve as fluorogenic substrates for ULPs that catalyze the release of fluorescent ACA (Figure 1B). Many ULPs are also DUBs. Eleven FLAG-UBL-GxC-6×His expression constructs, where x denotes the terminal glycine position, and UBL types include SUMO1–4, URM1, UFM1, NEDD8, ISG15, GABARAP, GABARAPL2, and MNSFβ were generated during previous research. Since the *N*-terminal FLAG tag is not expected to interfere with interactions with ULPs, we kept it in our study. Please note that ISG15, SUMO1–4, and MNSFβ natively contain a cysteine residue that may undergo the ACPL reaction as well. This cysteine was mutated to alanine in all six proteins. We followed the previously established protocols to express Ub-G76C-6×His and all 11 FLAG-UBL-GxC-6×His proteins. All proteins were then purified to above 90% purity, analyzed by SDS-PAGE, and shown in Figure S1A, before they were advanced to conduct the ACPL reaction with Gly-ACA. All proteins displayed a dimer band due to a disulfide bond formed between two monomers. We also subjected all purified proteins to electrospray ionization mass spectrometry (ESI-MS) analysis. Deconvoluted ESI-MS spectra, as shown in Figure S1B, confirmed their intact chemical compositions with molecular weights matching theoretical values (Figure S1B and Figures S2–S13). All purified proteins were then subjected to the ACPL reaction with Gly-ACA in the presence of NTCB (Figure 1C). In this one-pot reaction, 500 μM FLAG-UBL-GxC-6×His was mixed with 1 mM TCEP, 5 mM NTCB, and 500 mM Gly-ACA in 1× PBS buffer overnight at 37 °C. Gly-ACA was well soluble in these reaction conditions, allowing a 500 mM final concentration to be used. On the contrary, Gly-AMC could only achieve a final concentration of 40 mM. We set up a similar reaction to synthesize Ub-ACA as well by following our published protocol. All products were purified using FPLC and unreacted proteins were then removed by incubating with Ni-NTA resins. The purified probes were analyzed by SDS-PAGE (Figure S1C) and subjected to ESI-MS. For all 12 Ub/UBL-ACA products, their original proton-charged and deconvoluted spectra are presented in Figures S14–S25. Combined deconvoluted ESI-MS spectra for all 12 products are presented in Figure 1D as well. As shown in Figure 1D, all products have determined molecular weights that match closely with their theoretical values with a deviation of 0.3 Da, confirming their successful synthesis. We used Ub-ACA as an example to quantify the product yield that was determined as 26%. To assess whether the Ub-ACA conjugate retains native-like secondary structure, circular dichroism (CD) spectroscopy was performed. The CD spectrum of Ub-ACA closely resembled that of native ubiquitin (Figure S26), indicating that the overall protein fold is preserved following ACA conjugation. The ACPL strategy enabled one-pot, rapid and efficient generation of Ub/UBL-ACA without the need for intein, enzymatic conjugation or refolding steps, offering a straightforward approach to obtain fluorogenic ubiquitin substrates in a time and labor-efficient manner. This method significantly streamlines the preparation/purification process compared to traditional Ub-AMC synthesis.

With the successful synthesis of 12 Ub/UBL-ACAs, we proceeded to evaluate their utility as fluorogenic substrates for deubiquitinases (DUBs) or ULPs. For Ub-ACA, we tested its cleavage with a diverse panel of recombinant cysteine DUBs representing different mechanistic subclasses. These included UCHL1, UCHL3, UCHL5, USP2, USP5, USP7, USP9X, USP15, and USP21. Each DUB at 50 nM concentration was incubated with 400 nM Ub-ACA at pH 7.6, and enzymatic cleavage was monitored in real-time by measuring the release of the fluorescent ACA moiety. Results are presented in Figure 2A. All tested enzymes that belong to different classes exhibited robust activities to catalyze the release of fluorescent ACA from the fluorogenic Ub-ACA substrate, demonstrating Ub-ACA as a broadly applied substrate for DUBs.

**Figure 2:**
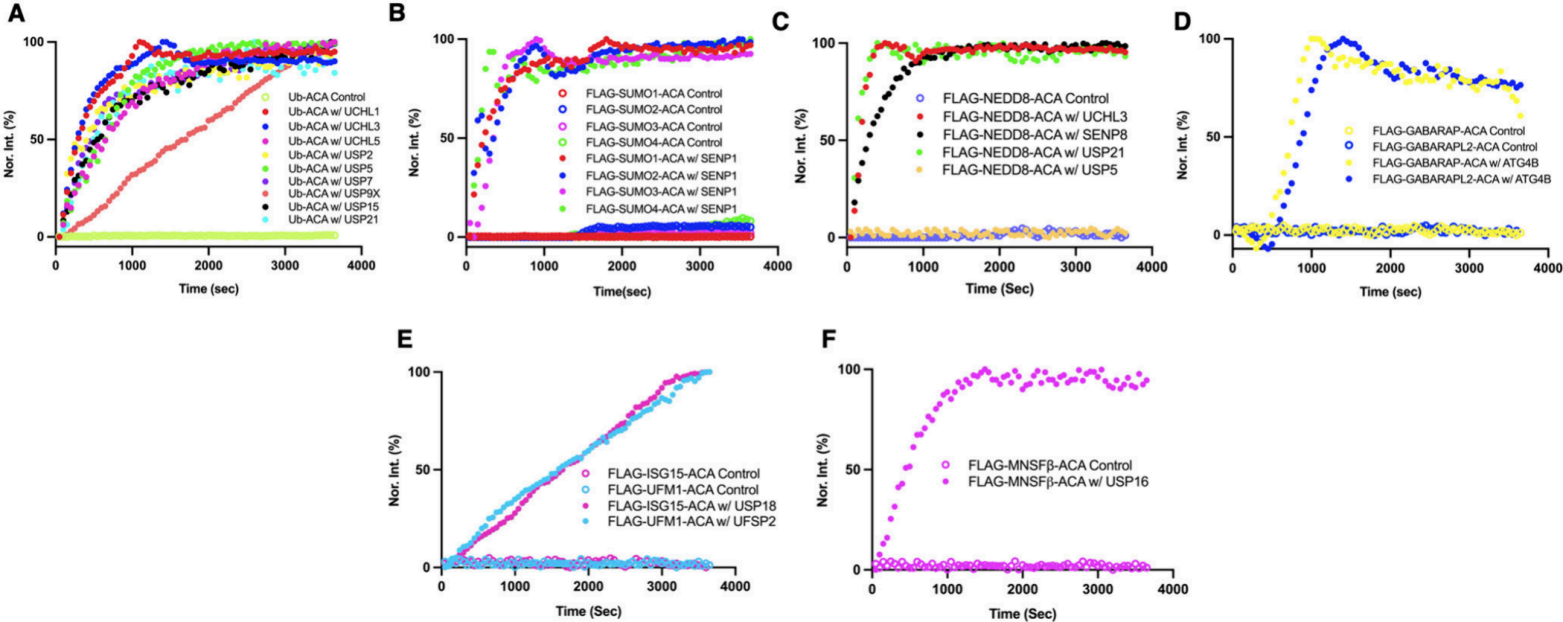
**Enzymatic cleavage of fluorogenic Ub/UBL-ACA substrates by recombinant DUBs and ULPs.** (**A**) Time-dependent fluorescence increase upon enzymatic cleavage of Ub-ACA by UCHL1, UCHL3, UCHL5, USP2, USP5, USP7, USP9X, USP15, and USP21. (B) SENP1 catalyzed processing of fluorogenic FLAG-SUMO1–4 ACA substrates. (C) Catalytic activities of UCHL3, SENP8, USP21, and USP5 toward FLAG-NEDD8-ACA. (**D**) Catalytic activities of ATG4B toward FLAG-GABARAP-ACA and FLAG-GABARAPL2-ACA. (E) Fluorescence emission upon enzymatic cleavage of FLAG-ISG15-ACA by USP18 and FLAG-UFM1-ACA by UFSP2. (**F**) Enzymatic cleavage of FLAG-MNSFβ-ACA by USP16. Reaction conditions: 50 nM for USP, UCH, SENP8 and UFSP2 enzymes, 100 pM for SENP1, 20 nM ATG4B with 400 nM substrate in assay buffer 50 mM Tris, 100 mM NaCl, 0.5 mM EDTA, 1 mM DTT, 0.1% BSA, pH 7.6.

The SENP family enzymes have been discovered as SUMO proteases.^21,22^ We acquired SENP1 and tested its activities on the four synthesized SUMO-ACA substrates. Reactions were set up similarly to those for Ub-ACA. As shown in Figure 2B, SENP1 displayed robust activities toward all four fluorogenic SUMO-ACA probes. Reactions for all four substrates finished within 500 s. Activity-based probes for SUMO proteases were previously developed.^23–25^ However, their applications in profiling SUMO proteases from human cells for proteomic characterizations have not been very successful. SENP1 was enriched but showed a quite low expression level. However, its broader substrate specificity and high catalytic efficiency toward all four SUMO isoforms may compensate for its low abundance. What is intriguing is its high activity toward SUMO4-ACA. Kinetic results indicate that while SENP1’s activity toward SUMO4 may be slightly higher than for the other three isoforms, all four SUMO-ACAs are cleaved with essentially comparable efficiencies. Compared to SUMO1–3, SUMO4 is a less abundant post-translational modification. It is also more structurally different from SUMO1–3, which have high sequence identity. It is likely that SENP1 involves recognition of commonly conserved regions in all four SUMO isoforms for almost equally high catalytic activities. This warrants further investigation. Compared to SUMO1–3, posttranslational SUMO4 modification is also less studied. Its efficient catalytic reversal by SENP1 indicates a highly regulatory mechanism. Further investigation on this aspect is needed as well. To validate the substrate specificity of our fluorogenic probes, we evaluated potential cross-reactivity between noncognate enzyme–substrate pairs. SENP1 was incubated with Ub-ACA, while UCHL1, a deubiquitinase, was tested with SUMO4-ACA. In both cases, no fluorescence signal was detected, indicating the absence of enzymatic cleavage (Figure S27). These results affirm that SENP1 does not process Ub-ACA and UCHL1 does not act on SUMO4-ACA, underscoring the substrate selectivity of the respective enzymes and supporting the specificity of SUMO4-ACA as a probe for SUMO proteases.

For NEDD8, SENP8 and UCHL3 are reported as its ULPs.^12,26^ Both of these enzymes showed a time-dependent increase in the fluorescence upon cleavage of FLAG-NEDD8-ACA (Figure 2C). Additionally, USP21 also showed robust enzymatic activity toward the probe. This observation reflects these enzymes’ capability to process NEDD8-conjugated substrates. Additionally, this result also reveals a dual substrate specificity for UCHL3 and USP21.^27^ This suggests that these enzymes possess the ability to accommodate structurally similar ubiquitin and NEDD8 moieties within their catalytic clefts. To evaluate the substrate specificity of NEDD8-ACA, we tested its reactivity with USP5, a deubiquitinase known to lack NEDD8 cross-reactivity. USP5 did not exhibit any detectable cleavage of NEDD8-ACA, thereby validating the specificity of the probe and underscoring its suitability as a selective substrate. ATG4B is a cysteine protease that plays a critical role in the autophagy pathway, a conserved cellular process responsible for degrading and recycling cytoplasmic components. It is known to process autophagy-related UBLs, including LC3 and GABARAP proteins. We tested its activities toward GABARAP-ACA and GABARAPL2-ACA. As shown in Figure 2D, both probes were efficiently cleaved by ATG4B. The kinetics exhibited a very interesting trajectory that clearly showed a two-phase process, with the first as a binding phase and the second as a catalytic phase. A substrate-binding triggered allosteric change for substrate-induced activation has been observed with ATG4B during its crystallography analysis.^28^ Our results are the first to show this allosteric change can be kinetically traced. Further studies in this aspect are needed.

USP18 is reported as a ULP for ISG15.^29^ We assessed the activity of USP18 using FLAG-ISG15-ACA. As shown in Figure 2E, cleavage of FLAG-ISG15-ACA by USP18 results in a strong fluorescence emission which further confirms USP18’s specificity toward ISG15-conjugated substrates. UFSP2 is known as a ULP for UFM1.^30^ We employed FLAG-UFM1-ACA to assess UFSP2’s catalytic activity. Upon cleavage by UFSP2, the release of ACA results in increased fluorescence (Figure 2E). This reaction confirms UFSP2’s role in processing UFM1 precursors and its specificity for UFM1 over other ubiquitin-like modifiers. USP16 has been reported as a ULP for MNSFβ.^31^ We conducted its activity analysis on FLAG-MNSFβ-ACA. Incubating USP16 with MNSFβ led to robust ACA cleavage as shown in Figure 2F, supporting that posttranslational MNSFβ modification is likely regulated by USP16.

We proceeded also to evaluate the use of all 12 Ub/UBL-ACAs as probes for DUBs and ULPs in human cell lysates, which represent a biologically relevant cellular environment. HEK293T cells were chosen for this purpose, given its most commonly used human cell line. We followed established protocols to culture, collect, and lyse HEK293T cells. Cell lysates with an overall protein load of 100 μg were then applied to incubation with all 12 Ub/UBL-ACAs at 400 nM concentration separately at 30 °C. Fluorescent release of ACA was monitored right away. As shown in Figure 3, all 12 Ub/UBL-ACAs were processed steadily by HEK293T cell lysates, indicating DUBs or UBLs exist for Ub and all 11 UBLs. Compared to Ub-ACA and SUMO1–4-ACAs that were completely hydrolyzed within 1 h, other UBLs were processed much more slowly, reflecting their lower abundance and less complicated roles in cells. To confirm that hydrolysis of Ub-ACA is mainly from cysteine DUBs, we did two further tests by pretreating HEK293T cell lysates with *N*-ethylmaleimide (NEM) to covalently block cysteine in cysteine enzymes or EDTA to sequester metal ions from metalloenzymes. These two pretreated cell lysates were then incubated with Ub-ACA to monitor ACA release, as shown in Figure 3A. NEM treatment completely inhibited Ub-ACA hydrolysis, indicating that cysteine DUBs are major Ub proteases in cells. EDTA-treated cell lysates showed a similar Ub-ACA hydrolysis trend. Figure 3B displayed cell lysate-catalyzed hydrolysis of four SUMO-ACAs, presenting a very similar trend. In combination with results from SENP1-catalyzed hydrolysis of four SUMO-ACAs shown in Figure 2B, we may cautiously conclude that four SUMOs may share similar regulatory mechanisms, such as ULPs.

**Figure 3:**
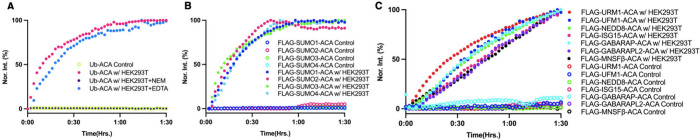
**DUB and ULP activities in HEK293T cell lysate toward (A) Ub-ACA, (B) FLAG-SUMO1–4, and (C) FLAG-URM1-ACA, FLAG-UFM1-ACA, FLAG-NEDD8-ACA, FLAG-ISG15-ACA, FLAG-GABARAP-ACA, FLAG-GABARAPL2-ACA, and FLAG-MNSFβ-ACA.** HEK293T cell lysates with a total protein load of 100 μg was incubated with 400 nM probe and Fluorescence ACA release was monitored right away for 90 min.

Figure 3C presents HEK293T cell lysate-catalyzed ACA release from less common UBLs. Their slow kinetics reflect their low abundance and, therefore, likely low abundance of regulatory enzymes existing in cells. However, unlike Ub and other UBLs, URM1 has no known ULPs. URM1 is also distinct from Ub and other UBLs in that it does not have a conventional E1-E2-E3 cascade. It is activated by an E1-like enzyme in both yeast and human cells to form a *C*-terminal thiocarboxylate that serves two roles, one to transfer sulfur for tRNA thiolation and the other for direct conjugation with substrate protein lysines. Our findings demonstrate that the URM1-ACA probe undergoes enzymatic cleavage in HEK293T cell lysate, indicating the presence of URM1-processing proteases in human cells. However, the identities of these proteases remain to be elucidated. To determine whether the enzymatic activity observed toward URM1-ACA in human cell lysates arises from cysteine-based enzymes, we performed an N-Ethylmaleimide (NEM) quenching assay. Pretreatment of HEK293T cell lysates with 1 mM NEM, a broad-spectrum cysteine protease inhibitor, completely abolished URM1-ACA cleavage activity (Figure S28). This result strongly suggests that the enzymatic processing of URM1-ACA is mediated by a cysteine-dependent protease. Since the identity of the putative deurmylase(s) remains unknown, the use of crude cell lysates was essential to capture endogenous activity and demonstrate that URM1-processing enzymes are indeed present in human cells. This cell-based strategy provides a functional readout in the absence of a defined recombinant enzyme, reinforcing the utility of URM1-ACA as a substrate to profile cysteine-based deurmylases in complex biological systems.

Overall, this study establishes Ub/UBL-ACAs as robust fluorogenic substrates for a broad spectrum of DUBs and ULPs. These probes, synthesized using the ACPL technique, maintain structural integrity at the *C*-terminus and mimic the native isopeptide linkage while offering a direct fluorescence readout upon enzymatic cleavage. Their compatibility with a wide range of DUBs and ULPs underscores their utility in real-time enzymatic profiling. Among the UBLs tested, SUMO4-ACA emerged as particularly interesting. Despite its structural difference from SUMO1–3, particularly in its N-terminal extension, SUMO4-ACA was processed by SENP1 and human cell lysates with about equal efficiency as SUMO1–3-ACA. This result suggests that the core recognition elements necessary for SUMO protease activities are conserved across all SUMO isoforms. SUMO4 has long been considered to serve cellular roles different from SUMO1–3. But our results indicate that it likely shares similar regulatory enzymes with SUMO1–3. Another interesting observation was made with ATG4B. It showed clearly a two-phase process that is kinetically traceable using our developed GABARAP-ACA and GABARAPL2-ACA probes, showcasing applications of these new probes in enzyme mechanistic analysis for DUBs and ULPs. The most intriguing observation in this study is the hydrolysis of URM1-ACA by human cell lysates, indicating the existence of deurmylase(s) in human cells. So far, there is no deurmylase reported in both yeast and human cells. Our results point to a validated direction for their identification. For the 12 probes described in this study they can be easily prepared using ACPL in a one-pot reaction setup, making it readily scalable. In drug discovery, for a long time, High-Throughput Screening (HTS) of DUBs against small molecule libraries using a Ub-based substrate was limited by the availability and affordability of fluorogenic Ub substrates. The Ub/UBL-ACA probes reported in this study will greatly facilitate DUB/ULP assays and potentially HTS, given their ease of synthesis.

## Supplementary Material



## Notes

Supplementary figures including SDS-PAGE and deconvoluted ESI-MS spectra for all purified Ub and UBL proteins, Ub/UBL-ACA probes, and supplementary table (PDF)
