# Supplementary material for: A Simple, Quick, and Scalable Route to Fluorogenic Ubiquitin and Ubiquitin-Like Protein Substrates for Assessing Activities of Deubiquitinases and Ubiquitin-Like Protein-Specific Proteases
Source: ACS Chem Biol. 2025 Aug 13;20(9):2075–80. doi: 10.1021/acschembio.5c00446 (PMC12455564; doi:10.1021/acschembio.5c00446)
Supplement: Supplementary file 1 [file cb5c00446_si_001.pdf]

Supplementary Information for

A Simple, Quick, and Scalable Route to Fluorogenic Ubiquitin and Ubiquitin-Like Protein Substrates for Assessing Activities of Deubiquitinases and Ubiquitin-Like Protein-Specific Proteases

Saibal Chanda[a], Alan Pham[b], Yifan Shi[b], Sandeep Atla[b], and Wenshe Ray Liu[a,b,c,d,e,*]

[a]Department of Biochemistry and Biophysics, College of Agriculture and Life Sciences, Texas A&M University, College Station, TX 77843, USA

[b]Texas A&M Drug Discovery Center and Department of Chemistry, College of Arts and Sciences, Texas A&M University, College Station, TX 77843, USA

[c]Institute of Biosciences and Technology and Department of Translational Medical Sciences, School of Medicine, Texas A&M University, Houston, TX 77030, USA

[d]Department of Cell Biology and Genetics, School of Medicine, Texas A&M University, College Station, TX 77843, USA

[e]Department of Pharmaceutical Sciences, Irma Lerma Rangel College of Pharmacy, Texas A&M University, College Station, TX 77843, USA

*Correspondence should be addressed to Wenshe Ray Liu: wsliu2007@tamu.edu

METHODS

Compounds. TCEP and NTCB were purchased from A2B chem LLC, San Diego, CA, USA.

Expression and Purification of UbG76C-6xHis Protein. $Ub_{1\text{-}75}$-Cys-6×His were recombinantly expressed and purified. An overnight culture of E. coli BL21(DE3) cells harboring a pETDuet-1 expression vector coding $Ub_{1\text{-}75}$-Cys-6×His was inoculated (1:100 dilution) into a 2xYT medium containing 100 µg/mL ampicillin. Cells were let grow at 37 ºC until OD600 reached 0.6-0.9 and 1 mM IPTG was added to induce protein expression. The cells were let grow in a shaker at 37 ºC for 8 h or 18 ºC for 16 h and collected by centrifugation (5,000 rpm, 30 min, 4 ºC). The cell pellets were resuspended in a 100 mL lysis buffer (50 mM NaH_2PO4, 500 mM NaCl, 5 mM imidazole, 1 mM TCEP, pH 7.8) and lysed by sonication. The cell lysates were clarified by centrifugation (10,000 rpm, 30 min, 4 ºC), and the supernatant was decanted. 6 M HCl solution was then added dropwise into the supernatant to adjust pH to 2 to drive the formation of white precipitate inside the solution. The precipitate was pelleted by centrifugation (10,000 rpm, 30 min, 4 ºC) and removed. The pH value of the supernatant was then adjusted back to 7.8 by adding 6 M NaOH. This supernatant was loaded onto high-affinity Ni^{2+}-NTA resins at RT and then washed with a buffer (50 mM NaH_2PO_4, 500 mM NaCl, 30 mM imidazole, 1 mM TCEP, pH 7.8). The bound protein was eluted by another buffer (50 mM NaH_2PO_4, 250 mM NaCl, 300 mM imidazole, 1 mM TCEP, pH 7.8) and then desalted into ammonium bicarbonate (ABC) buffer using HiPrep 26/10 Desalting column (Cytiva). All proteins were concentrated using 3 kDa MWCO Amicon ultracentrifugation filter devices (Millipore Sigma), and concentrations were measured using a NanoDrop spectrophotometer (ThermoFisher). Eventually, all proteins were aliquoted, lyophilized, and stored at -80 ºC.

Expression and Purification of FLAG-UBL-GxC-6xHis Proteins. An overnight culture of E. coli BL21(DE3) cells harboring a Ubl expression vector was inoculated (1:100 dilution) into 2xYT medium containing 100 µg/mL ampicillin. Cells were let grow at 37 ºC until OD600 reached 0.6-0.9 and then 1 mM IPTG was added to induce protein expression. Induced cells were let grow in a refrigerated incubating shaker at 18 ºC overnight and harvested by centrifugation (4000 rpm, 20 min, 4 ºC). The cell pellet was then lysed, purified by Ni^{2+}-NTA resins, and desalted as same as mentioned previously for Ub protein. Eventually, the proteins were aliquoted, lyophilized, and stored at -80 ºC.

Use of ACPL to Synthesize Ub/UBL-ACA Fluorogenic Probes. A 500 mM TCEP stock solution was prepared in water, and a 500 mM NTCB stock solution was prepared in DMSO. 500mM of GlyACA was dissolved into in 250µL of 1× PBS buffer, pH-7.4, and pH was slowly adjusted to 9.5 using 6M NaOH. At this stage, Ub/UBL protein pellets were dissolved in the solution, followed by sequential addition of 1µL TCEP (from 500mM stock) and 5µL NTCB (from 500mM stock). Make up the volume to 500µL using 1× PBS buffer. The reaction mixtures were incubated at 37 ºC overnight, desalted using a HiTrap column, and then incubated with Ni^{2+}-NTA resins for 30mins at 4ºC and were removed with micro syringe filters, and the flowthroughs were collected for ESI-MS analysis.

Preparation of HEK293T cell lysate. HEK293T cells were cultured in DMEM (Gibco) supplemented with 10% fetal bovine serum (FEB, Gibco) and 5% penicillin-streptomycin (Pen-Strep, Sigma; Pen: 10,000 units/Strep: 10 mg/mL). The cells were grown in T75 flasks in a 37 ºC, 5% CO_2 incubator until they reached approximately 90% confluency. Subsequently, the cells were collected in 1× PBS buffer and transferred into 1.5 mL tubes. To lyse the cells, Pierce (ThermoFisher) IP protease-free cell lysis buffer was added to the cell suspension. The mixture was gently shaken under refrigeration for 30 min to ensure cell lysis. After the cell lysis process, the cell lysate was clarified by centrifugation at 14,000 rpm for 30 min at 4 ºC. The resulting supernatant was collected, and the protein concentration was measured by the BCA assay.

Monitoring Enzymatic Activity in HEK293T cell lysate using Ub/UBL-ACA probes. In a 96-well plate, 50µL of HEK293T cell lysate with a total protein load of 100µg was mixed with 50µL assay buffer containing

50mM Tris, 100mM NaCl, 0.5mM EDTA, 1mM DTT, 0.1% BSA (pH-7.6). The mixture was incubated for 5mins 30 °C. 400nM of each Ub/UBL-ACA probes were added separately, and fluorescence measurements were taken at 30 °C for 1.5 hours at every 2-minute interval (excitation at 380 nm and emission at 460 nm). At each time point, the measured fluorescence intensity was baseline-corrected by subtracting the mean fluorescence of the blank control. The corrected values were then normalized by dividing by the difference between the maximum fluorescence intensity and the mean blank control fluorescence.

Monitoring the catalytic activity of DUBs/ULPs using Ub/UBL-ACA probes. In a 96-well plate, 1µL USP and UCH enzymes (from a 5µM stock prepared in 1× PBS buffer), 1µL SENP1 (from 10nM stock prepared in 1× PBS buffer), 2µL ATG4B (from 1µM stock prepared in 1× PBS buffer) were added (final concentration for USP, UCH, SENP8, UFSP2 was 50nM, 100pM for SENP1, 20nM for ATG4B) in 100µL assay buffer containing 50mM Tris, 100mM NaCl, 0.5mM EDTA, 1mM DTT, 0.1% BSA (pH-7.6), and incubated at 30 °C for 5mins. 400nM of each Ub/UBL-ACA probes were added separately, and fluorescence measurements were taken 30 °C for 1 hour at every 50-second interval (excitation at 380 nm and emission at 460 nm). At each time point, the measured fluorescence intensity was baseline-corrected by subtracting the mean fluorescence of the blank control. The corrected values were then normalized by dividing by the difference between the maximum fluorescence intensity and the mean blank control fluorescence.

N-Ethylmaleimide (NEM) quenching assay using FLAG-URM1-ACA. HEK293T cell lysates were incubated with 1mM NEM at 4 °C for 2-3 hours. After that, in a 96-well plate, 50µL of NEM pre-treated HEK293T cell lysate with a total protein load of 100µg was mixed with 50µL assay buffer containing 50mM Tris, 100mM NaCl, 0.5mM EDTA, 1mM DTT, 0.1% BSA (pH-7.6). The mixture was incubated for 5mins 30 °C. Appropriate control samples were also set up. 400nM of FLAG-URM1-ACA probe was added separately, and fluorescence measurements were taken at 30 °C for 1.5 hours at every 2-minute interval (excitation at 380 nm and emission at 460 nm). At each time point, the measured fluorescence intensity was baseline-corrected by subtracting the mean fluorescence of the blank control. The corrected values were then normalized by dividing by the difference between the maximum fluorescence intensity and the mean blank control fluorescence.

Supplementary Figures

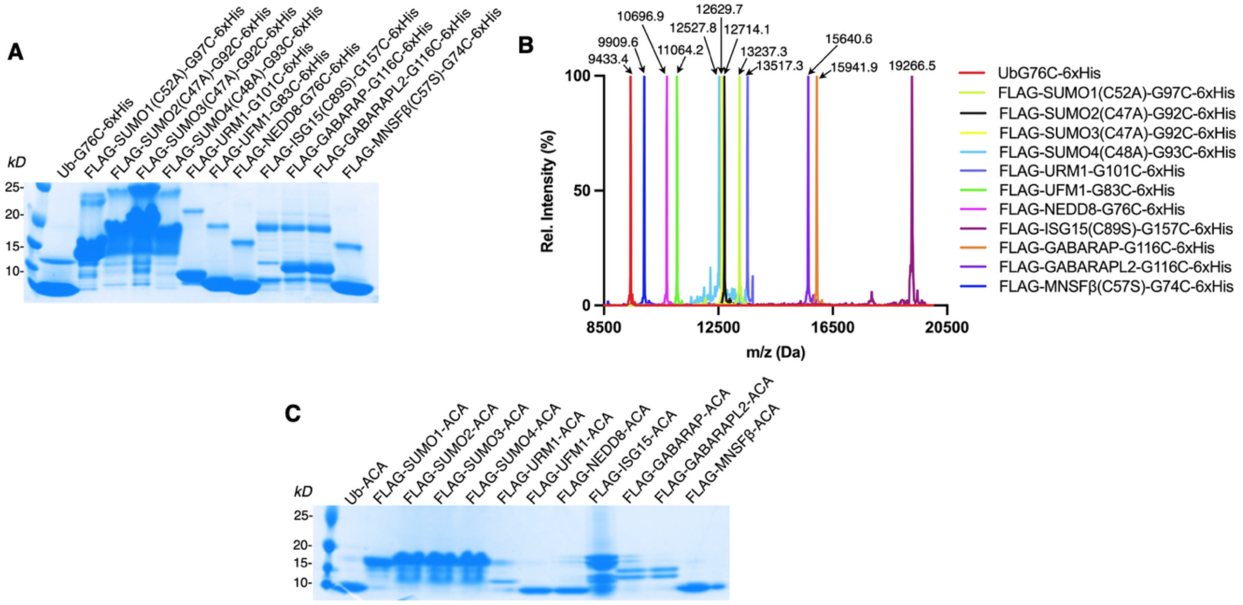

Figure S1. (**A**) SDS-PAGE and Coomassie blue staining of Ub$_{1-75}$-Cys-6×His, FLAG-SUMO1(C52A)-G97C-6×His, FLAG-SUMO2(C48A)-G93C-6×His, FLAG-SUMO3(C47A)-G92C-6×His, FLAG-SUMO4(C48A)-G93C-6×His, FLAG-URM1-G101C-6×His, FLAG-UFM1-G83C-6×His, FLAG-NEDD8-G76C-6×His, FLAG-ISG15(C89S)-G157C-6×His, FLAG-GABARAP-G116C-6×His, FLAG-GABARAPL2-G116C-6×His, and FLAG-MNSFβ(C57S)-G74C-6×His; (**B**) Deconvoluted ESI-MS spectra of the proteins mentioned in A. Arrow-indicated values represent detected molecular weights of corresponding proteins. (**C**) SDS-PAGE and Coomassie blue staining of synthesized ACA probes: Ub-ACA, FLAG-SUMO1-ACA, FLAG-SUMO2-ACA, FLAG-SUMO3-ACA, FLAG-SUMO4-ACA, FLAG-URM1-ACA, FLAG-UFM1-ACA, FLAG-NEDD8-ACA, FLAG-ISG15-ACA, FLAG-GABARAP-ACA, FLAG-GABARAPL2-ACA, and FLAG-MNSFβ-ACA.

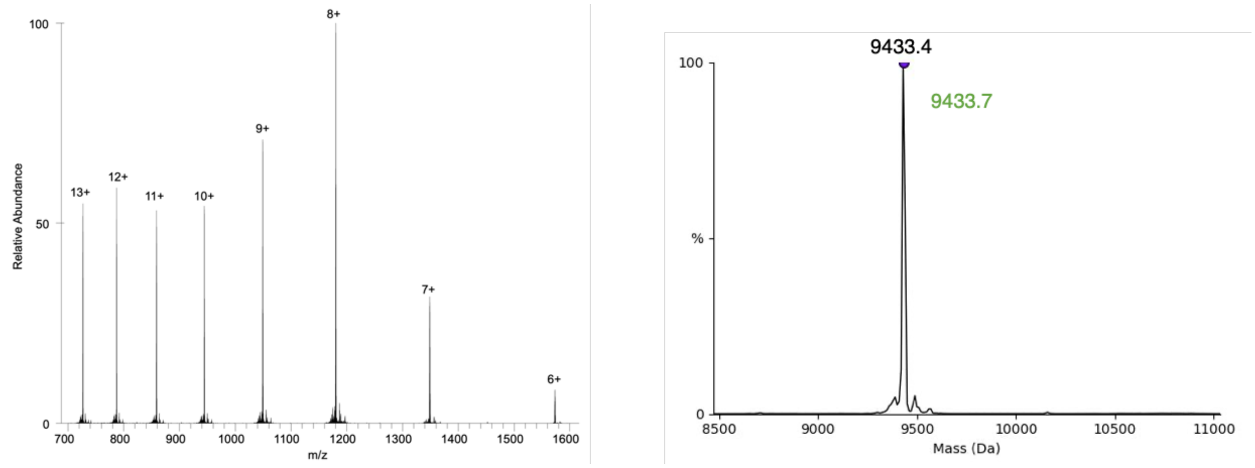

Figure S2. ESI-MS spectra and deconvoluted mass of Ub_{1-75}-Cys-6×His. The value in green indicates the actual theoretical mass of the protein.

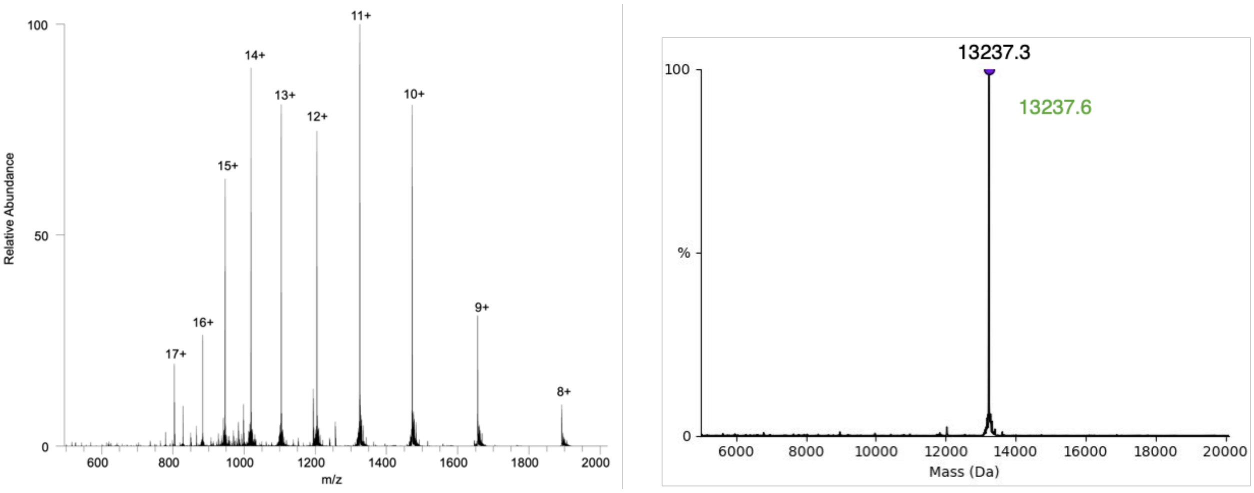

Figure S3. ESI-MS spectra and deconvoluted mass of FLAG-SUMO1(C52A)-G97C-6×His. The value in green indicates the actual theoretical mass of the protein.

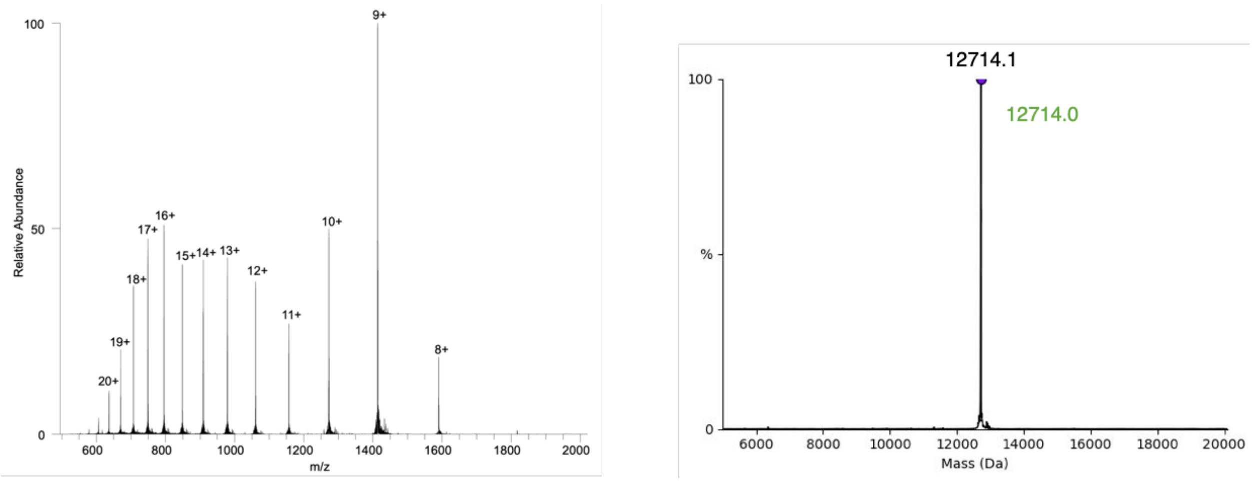

Figure S4. ESI-MS spectra and deconvoluted mass of FLAG-SUMO2(C48A)-G93C-6×His. The value in green indicates the actual theoretical mass of the protein.

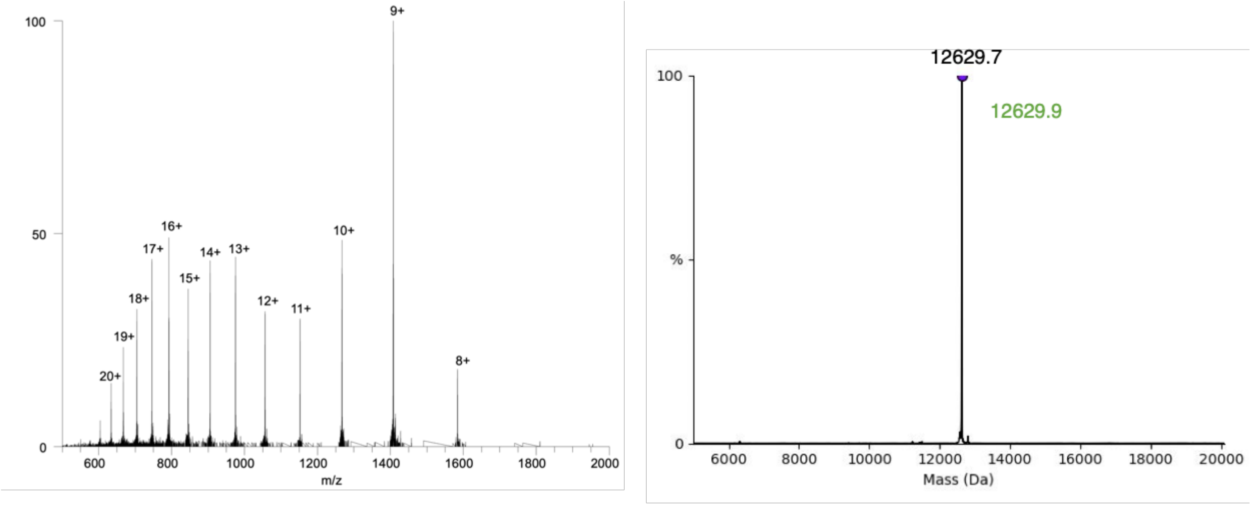

Figure S5. ESI-MS spectra and deconvoluted mass of FLAG-SUMO3(C47A)-G92C-6×His. The value in green indicates the actual theoretical mass of the protein.

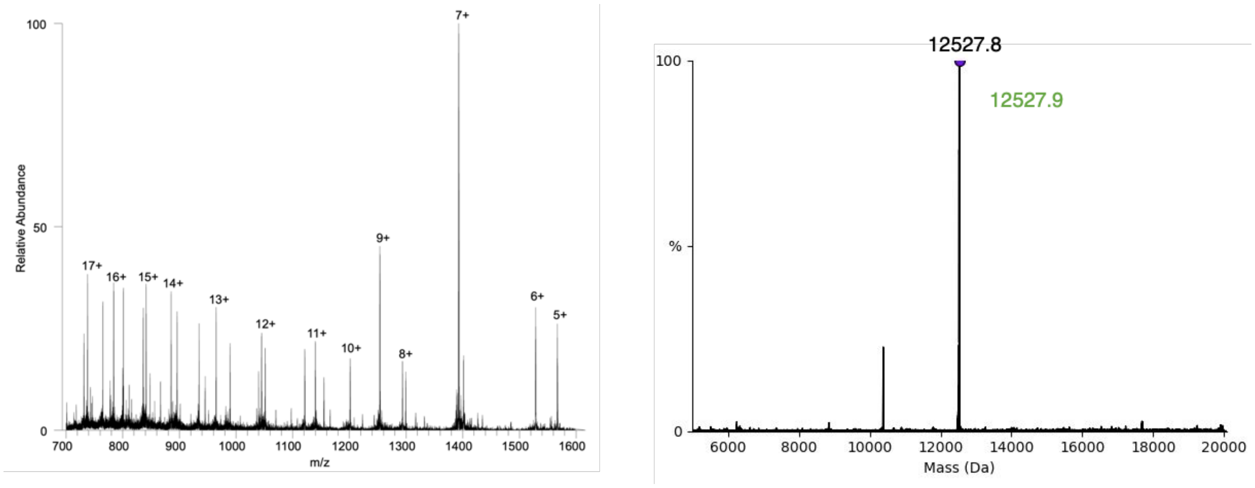

Figure S6. ESI-MS spectra and deconvoluted mass of FLAG-SUMO4(C48A)-G93C-6×His. The value in green indicates the actual theoretical mass of the protein.

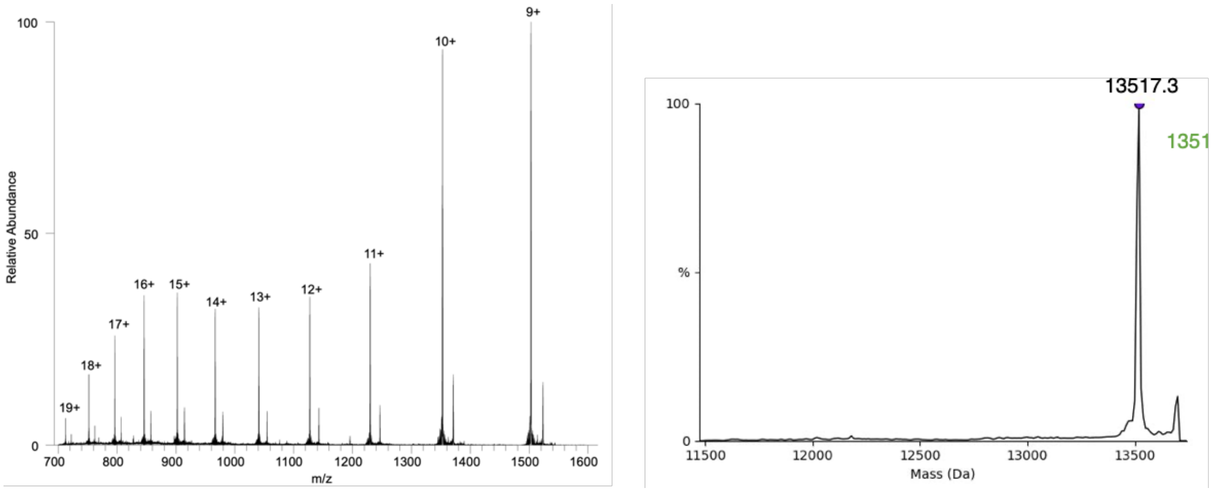

Figure S7. ESI-MS spectra and deconvoluted mass of FLAG-URM1-G101C-6×His. The value in green indicates the actual theoretical mass of the protein.

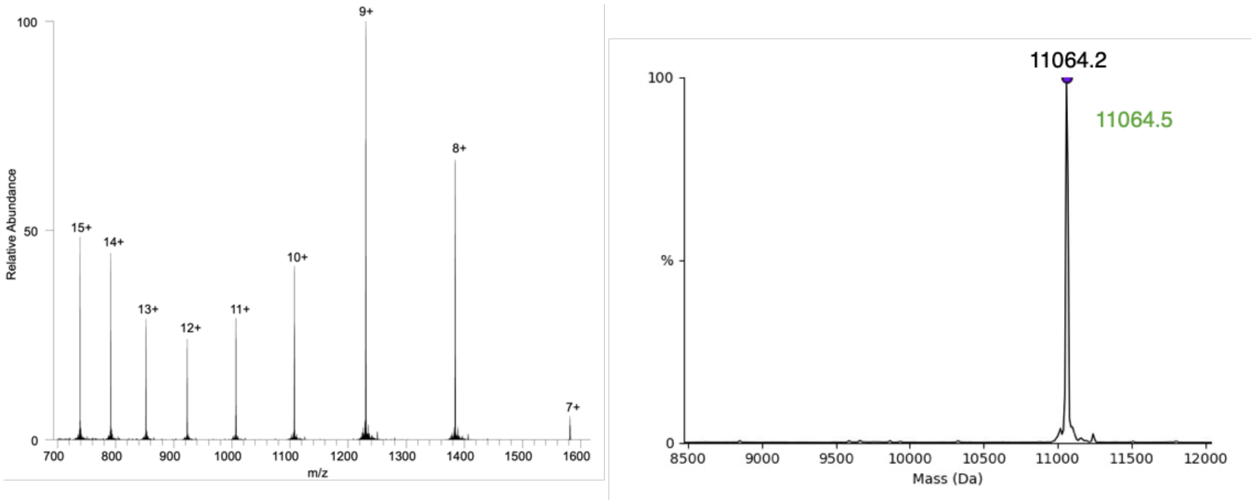

Figure S8. ESI-MS spectra and deconvoluted mass of FLAG-UFM1-G83C-6×His. The value in green indicates the actual theoretical mass of the protein.

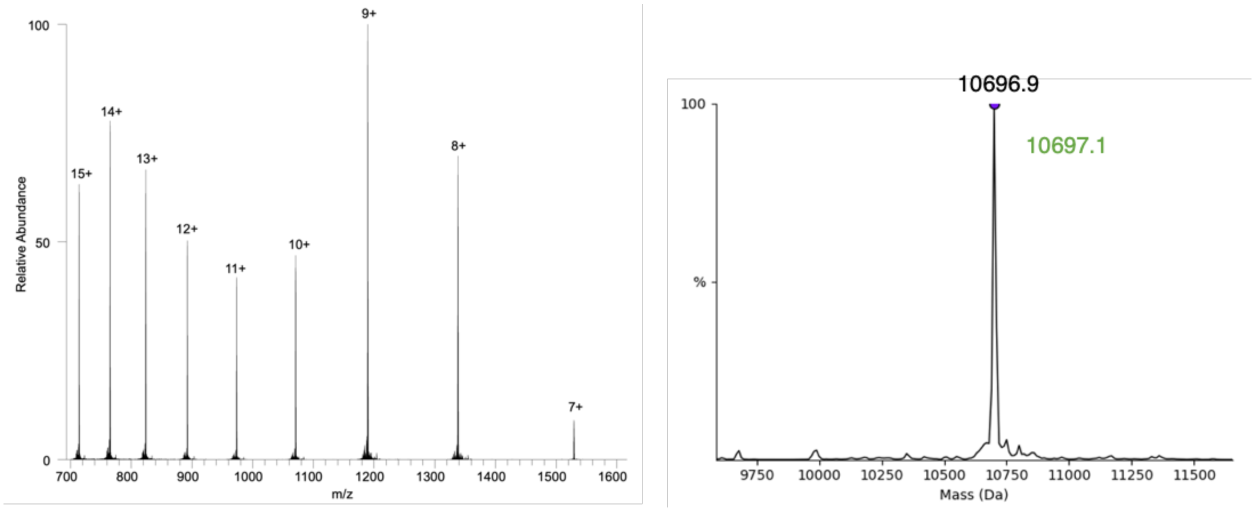

Figure S9. ESI-MS spectra and deconvoluted mass of FLAG-NEDD8-G76C-6×His. The value in green indicates the actual theoretical mass of the protein.

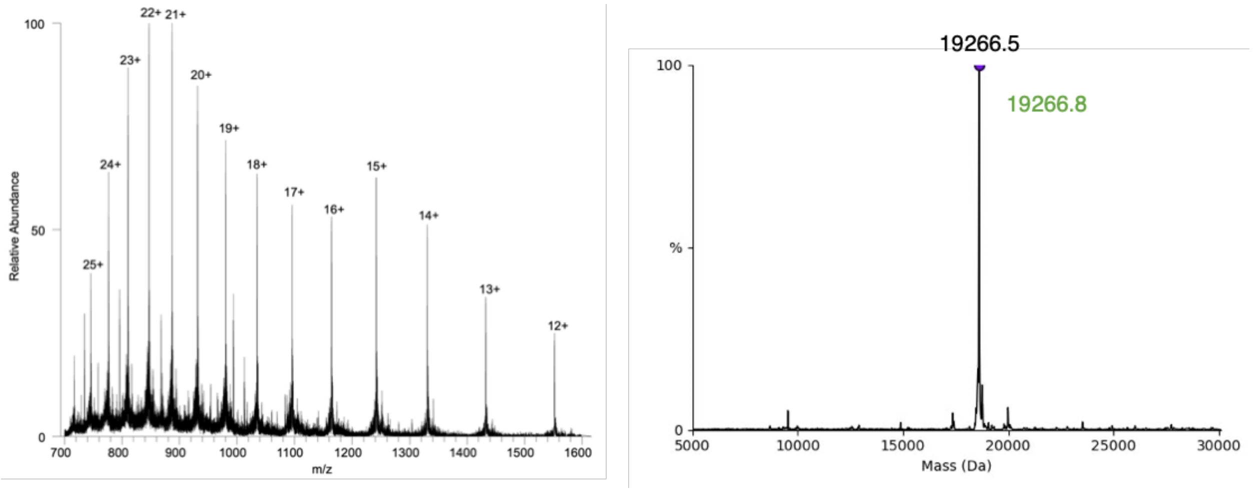

Figure S10. ESI-MS spectra and deconvoluted mass of FLAG-ISG15(C89S)-G157C-6×His. The value in green indicates the actual theoretical mass of the protein.

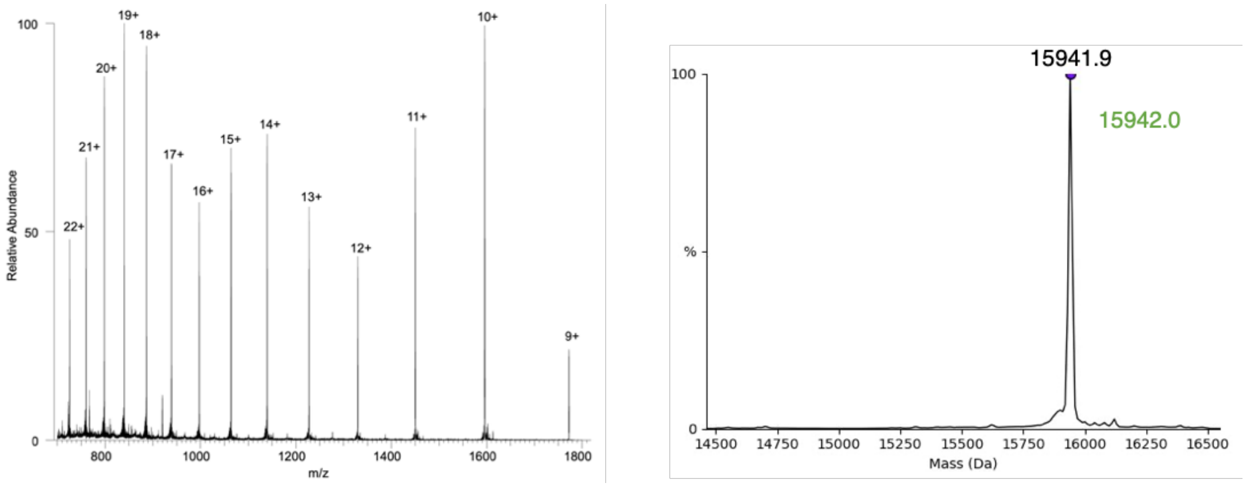

Figure S11. ESI-MS spectra and deconvoluted mass of FLAG-GABARAP-G116C-6×His. The value in green indicates the actual theoretical mass of the protein.

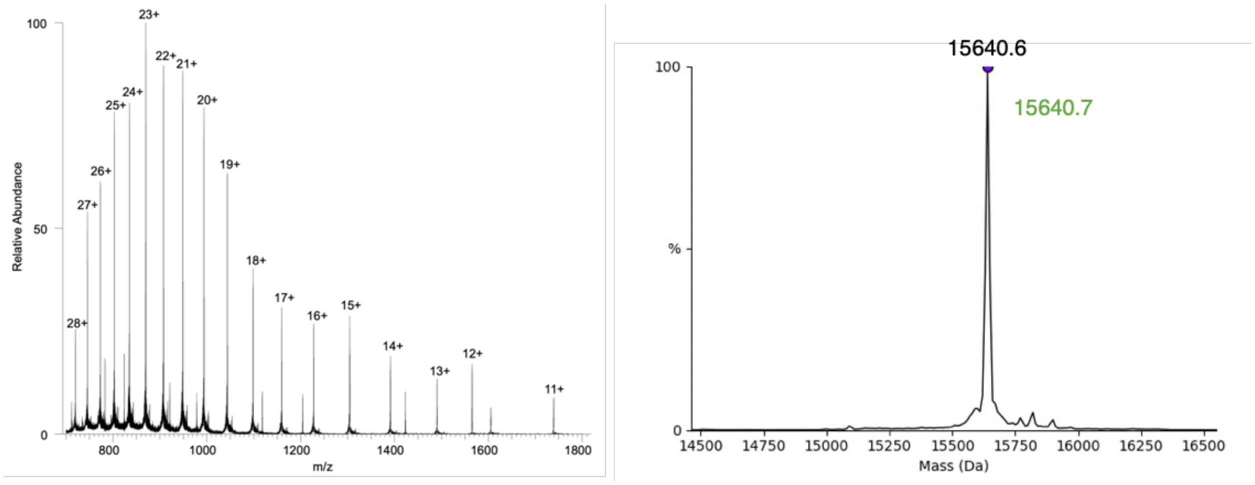

Figure S12. ESI-MS spectra and deconvoluted mass of FLAG-GABARAPL2-G116C-6×His. The value in green indicates the actual theoretical mass of the protein.

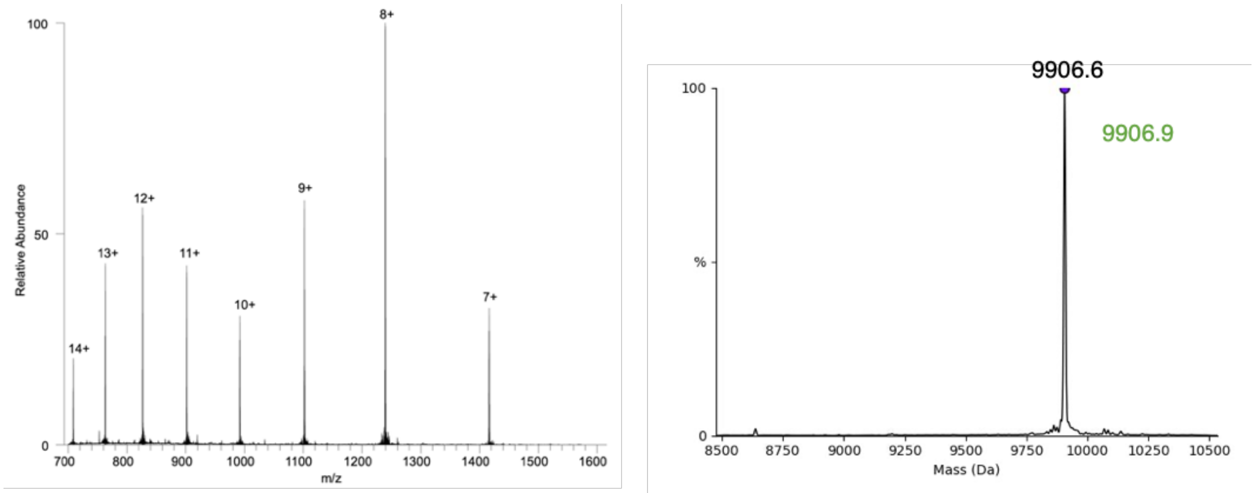

Figure S13. ESI-MS spectra and deconvoluted mass of FLAG-MNSFβ(C57S)-G74C-6×His. The value in green indicates the actual theoretical mass of the protein.

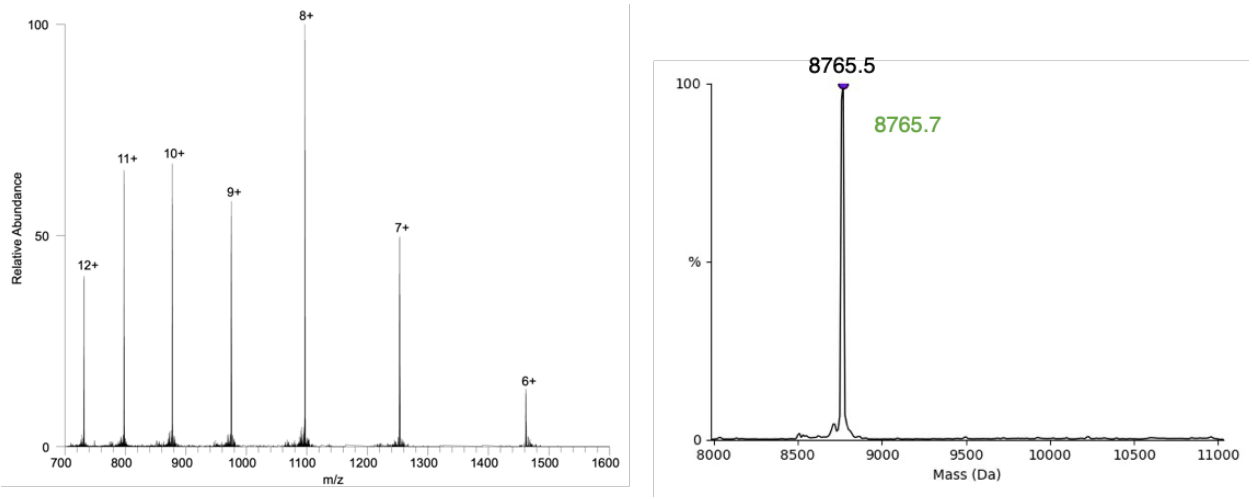

Figure S14: ESI-MS spectra and deconvoluted mass of Ub-ACA. The value in green indicates the actual theoretical mass of the probe.

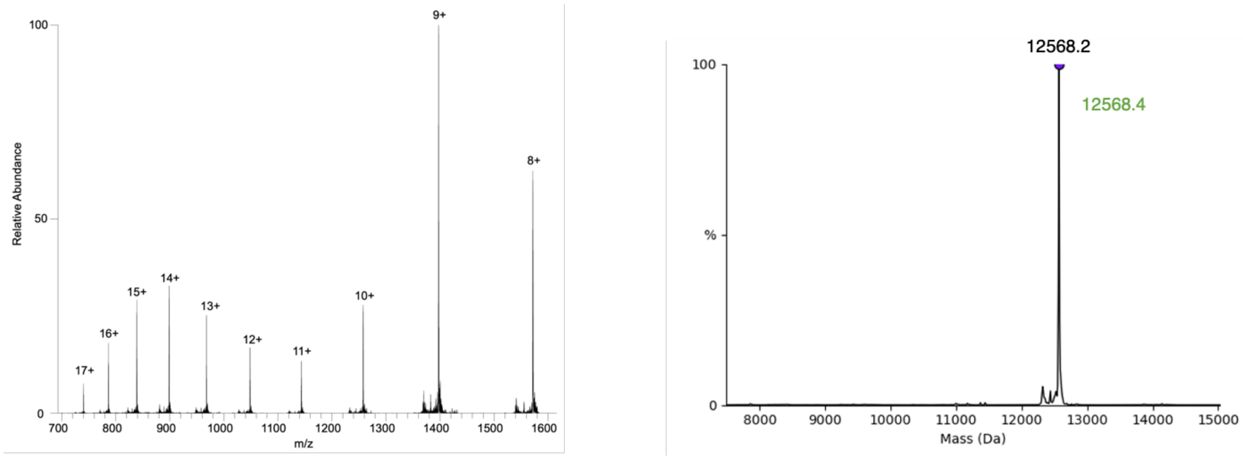

Figure S15: ESI-MS spectra and deconvoluted mass of FLAG-SUMO1-ACA. The value in green indicates the actual theoretical mass of the probe.

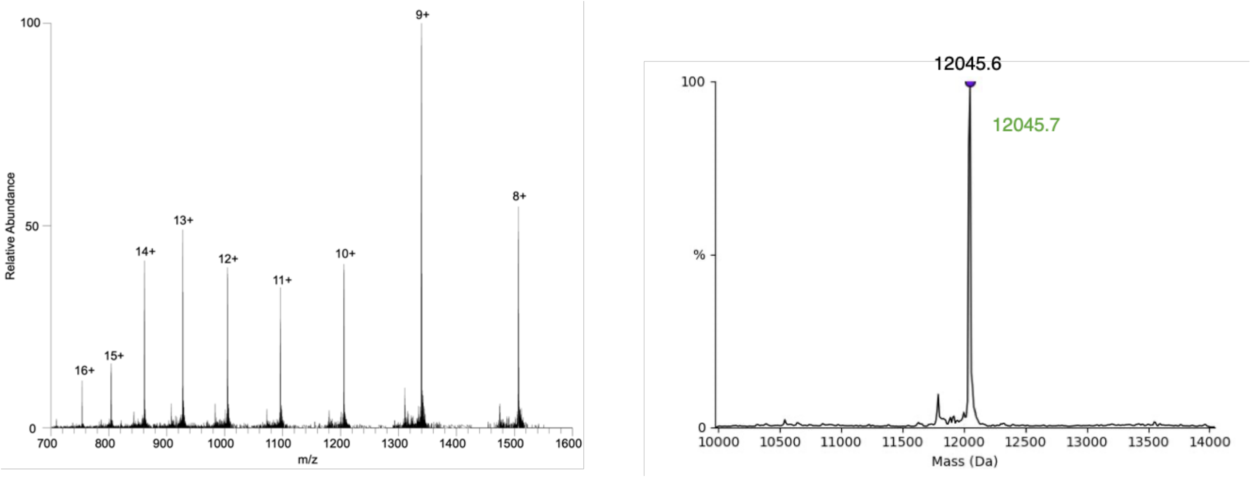

Figure S16: ESI-MS spectra and deconvoluted mass of FLAG-SUMO2-ACA. The value in green indicates the actual theoretical mass of the probe.

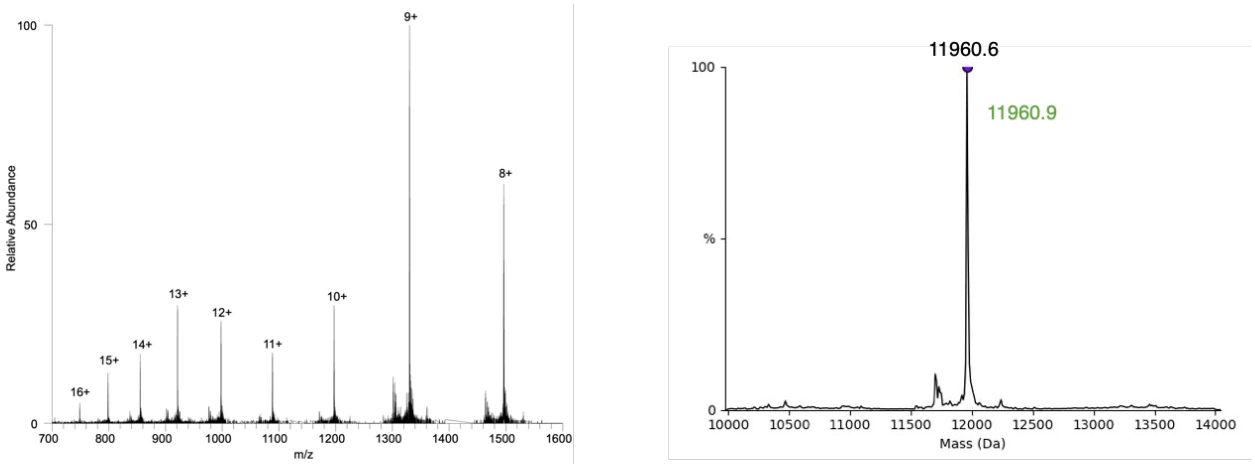

Figure S17: ESI-MS spectra and deconvoluted mass of FLAG-SUMO3-ACA. The value in green indicates the actual theoretical mass of the probe.

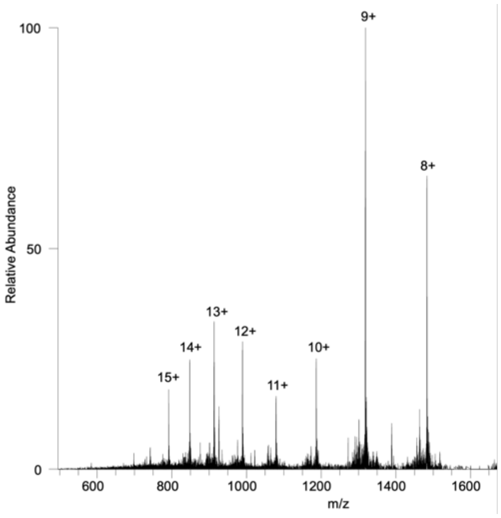

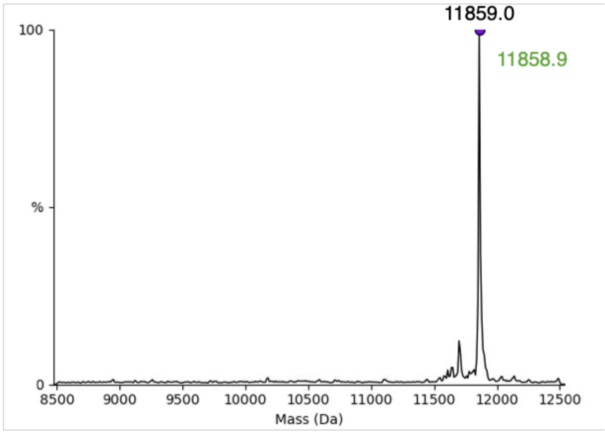

Figure S18: ESI-MS spectra and deconvoluted mass of FLAG-SUMO4-ACA. The value in green indicates the actual theoretical mass of the probe.

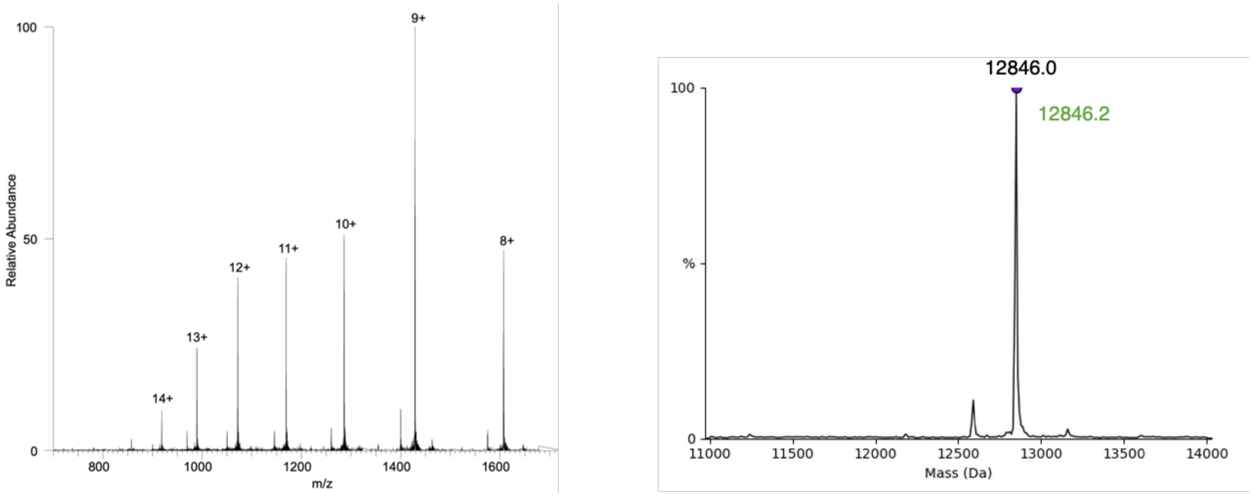

Figure S19: ESI-MS spectra and deconvoluted mass of FLAG-URM1-ACA. The value in green indicates the actual theoretical mass of the probe.

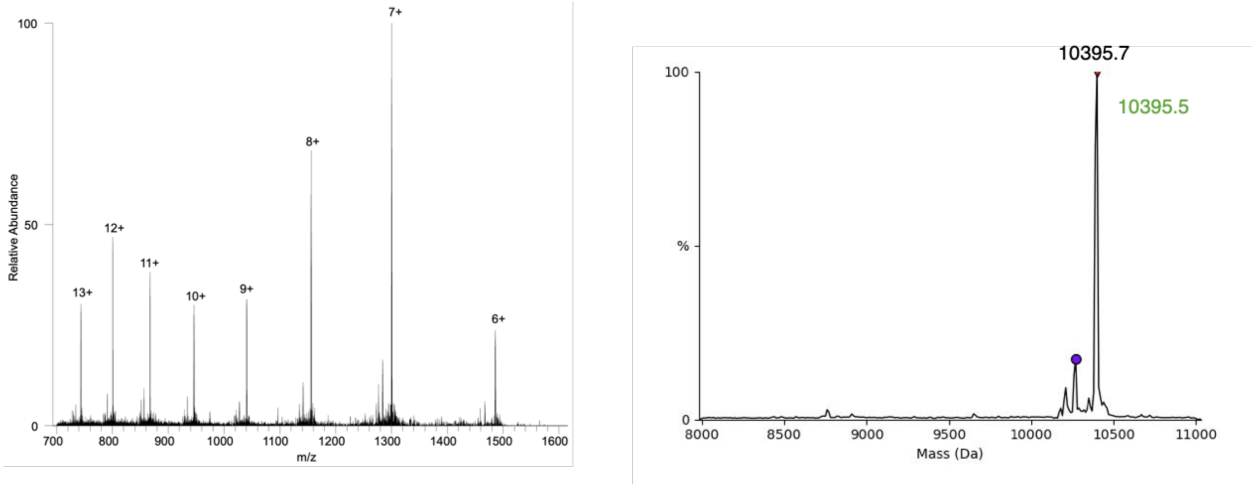

Figure S20: ESI-MS spectra and deconvoluted mass of FLAG-UFM1-ACA. The value in green indicates the actual theoretical mass of the probe.

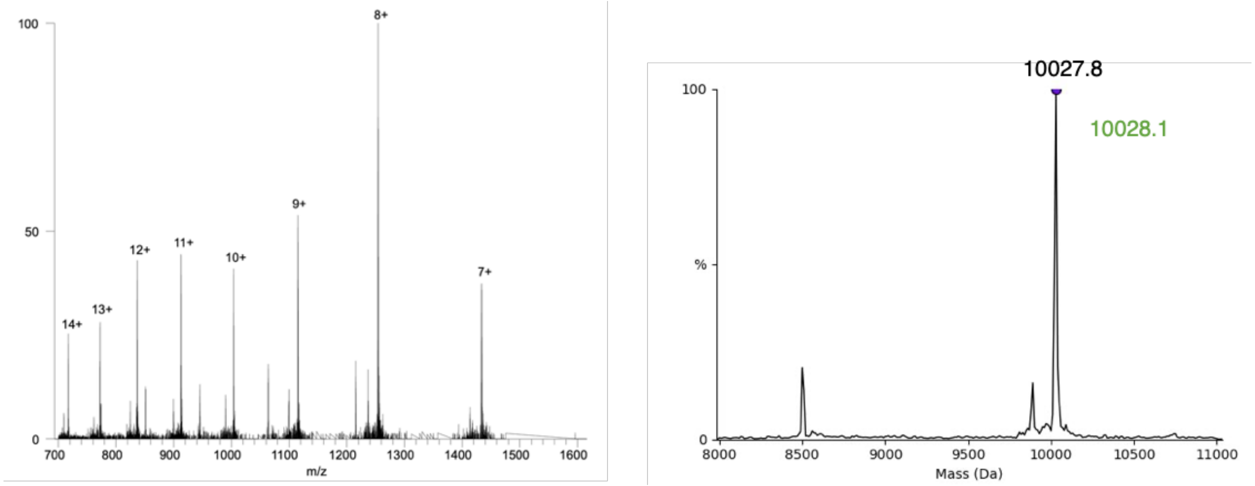

Figure S21: ESI-MS spectra and deconvoluted mass of FLAG-NEDD8-ACA. The value in green indicates the actual theoretical mass of the probe.

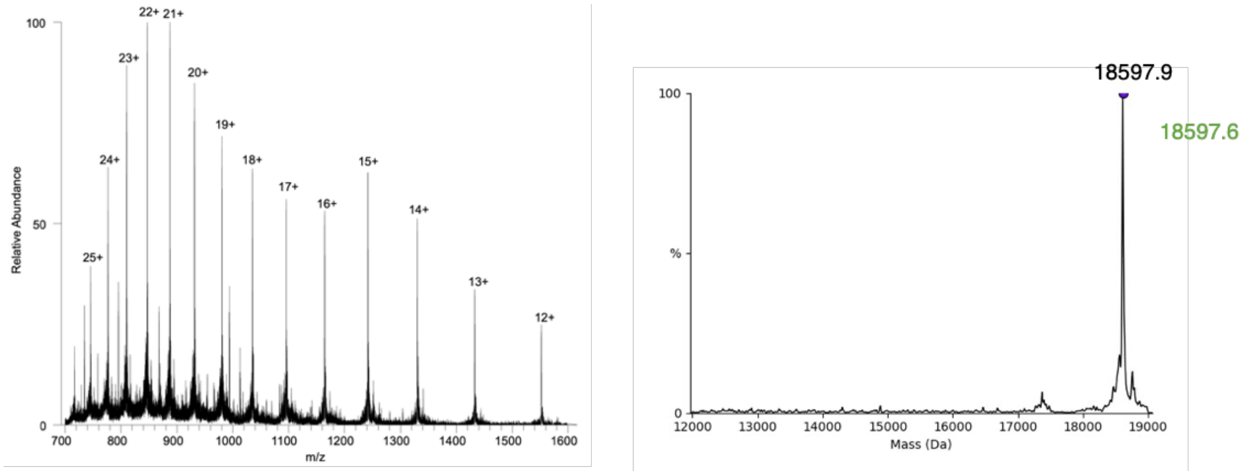

Figure S22: ESI-MS spectra and deconvoluted mass of FLAG-ISG15-ACA. The value in green indicates the actual theoretical mass of the probe.

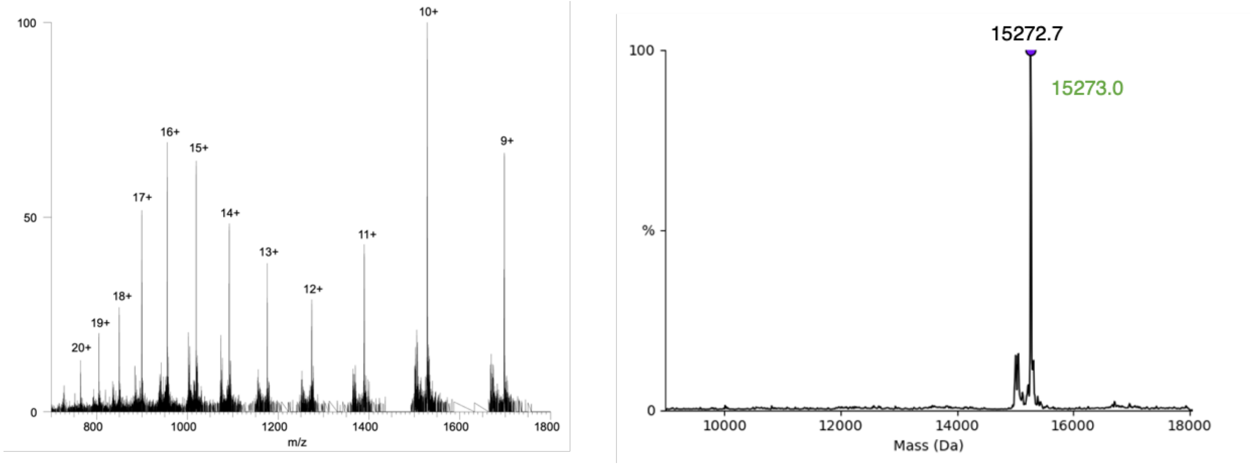

Figure S23: ESI-MS spectra and deconvoluted mass of FLAG-GABARAP-ACA. The value in green indicates the actual theoretical mass of the probe.

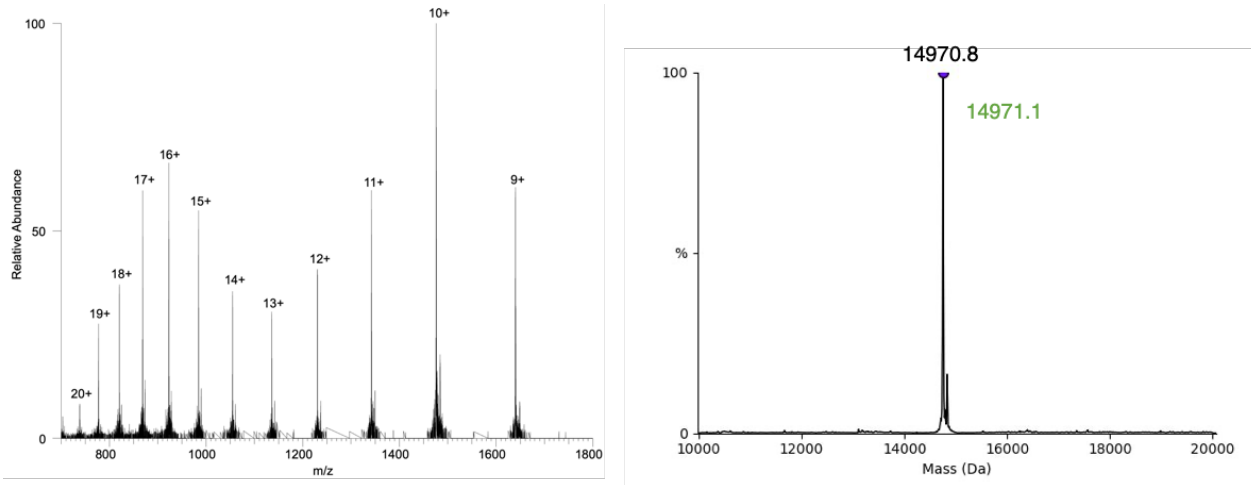

Figure S24: ESI-MS spectra and deconvoluted mass of FLAG-GABARAPL2-ACA. The value in green indicates the actual theoretical mass of the probe.

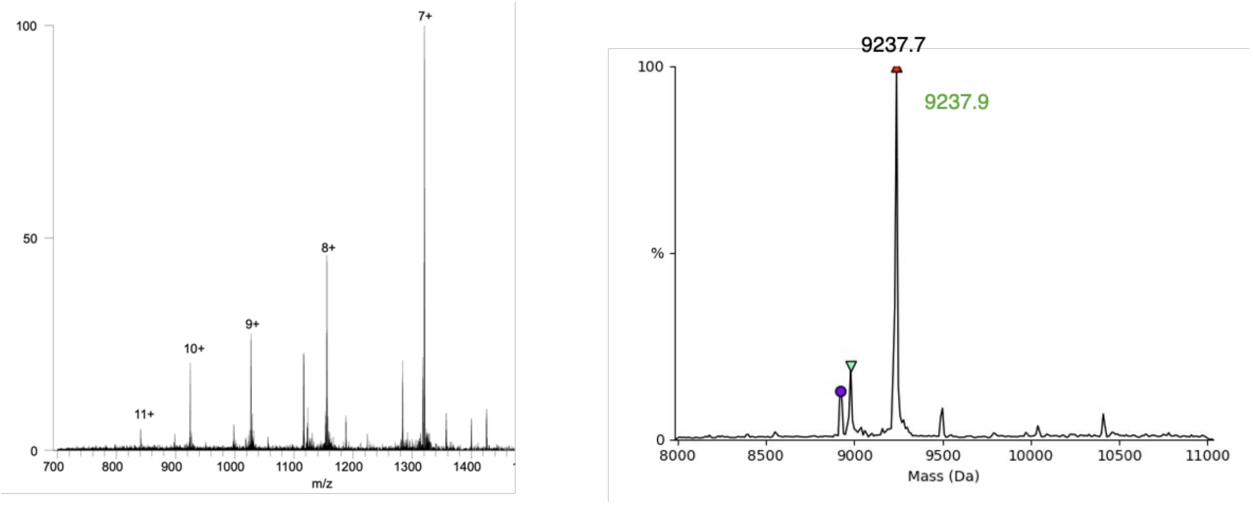

Figure S25: ESI-MS spectra and deconvoluted mass of FLAG-MNSFβ-ACA. The value in green indicates the actual theoretical mass of the probe.

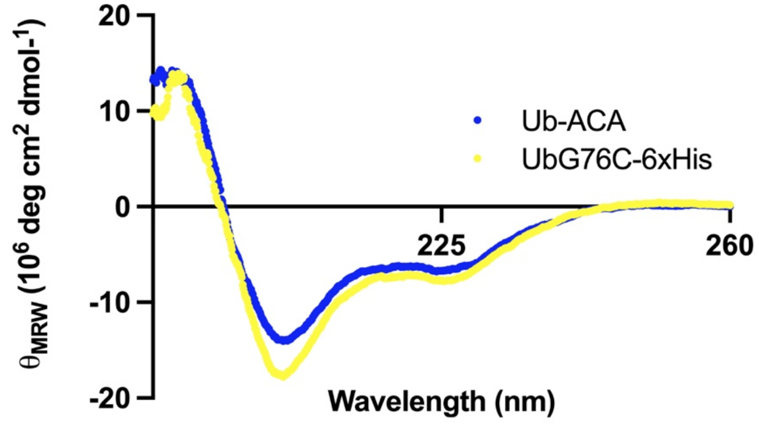

Figure S26: CD spectra of Ub-ACA and Ub76C-6xHis which shows that Ub-ACA retains a secondary structure consistent with UbG76C-6xHis, indicating proper folding after the ACPL reaction.

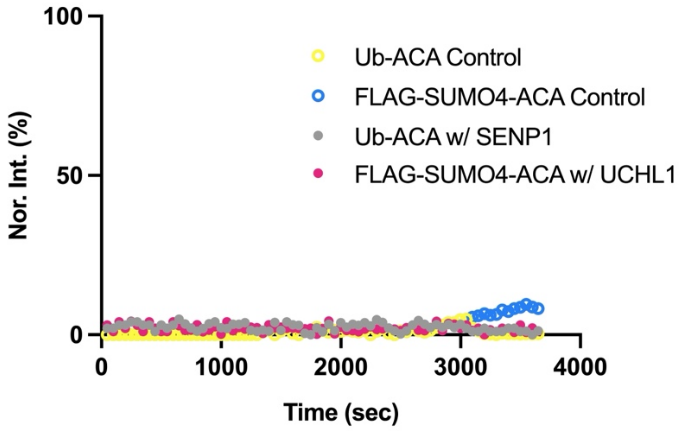

Figure S27: **Assessment of cross-reactivity between FLAG-SUMO4-ACA and Ub-ACA with non-cognate proteases.** Fluorescence assay showing that SENP1 does not cleave Ub-ACA, indicating lack of cross-reactivity between a SUMO protease and a ubiquitin-derived substrate. UCHL1 fails to cleave FLAG-SUMO4-ACA, confirming the substrate selectivity of this deubiquitinase. Lack of signal increase indicates no detectable enzymatic activity under the tested conditions.

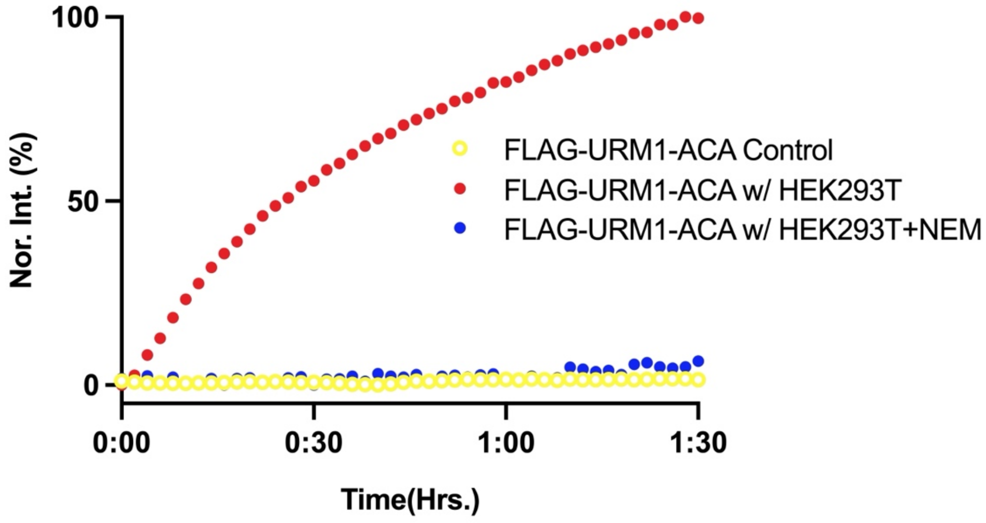

Figure S28: **Inhibition of FLAG-URM1-ACA cleavage by N-Ethylmaleimide (NEM) in HEK293T cell lysates.** HEK293T lysates were pretreated with or without 1mM NEM before incubation with FLAG-URM1-ACA. The loss of fluorescence signal upon NEM treatment indicates that URM1-ACA processing is mediated by endogenous cysteine-dependent enzymes, supporting the presence of putative deurmylases in human cells.

Table S1. The molecular weight of Ub/Ubls and synthesized Ub/Ubl-ACA probes

Proteins & Probes	Observed mass (Da)	Theoretical mass (Da)
Ub_{1-75}-Cys-6×His	9433.4	9433.7
Ub-ACA	8765.5	8765.7
FLAG-SUMO1(C52A)-G97C-6×His	13237.3	13237.6
FLAG-SUMO1-ACA	12568.2	12568.4
FLAG-SUMO2(C48A)-G93C-6×His	12714.1	12714.0
FLAG-SUMO2-ACA	12045.6	12045.7
FLAG-SUMO3(C47A)-G92C-6×His	12629.7	12629.9
FLAG-SUMO3-ACA	11960.6	11960.9
FLAG-SUMO4(C48A)-G93C-6×His	12527.8	12527.9
FLAG-SUMO4-ACA	11859.0	11858.9
FLAG-URM1-G101C-6×His	13517.3	13517.2
FLAG-URM1-ACA	12846.0	12846.2
FLAG-UFM1-G83C-6×His	11064.2	11064.5
FLAG-UFM1-ACA	10395.7	10395.5
FLAG-NEDD8-G76C-6×His	10696.9	10697.1
FLAG-NEDD8-ACA	10027.8	10028.1
FLAG-ISG15(C89S)-G157C-6×His	19266.5	19266.8
FLAG-ISG15-ACA	18597.9	18597.6
FLAG-GABARAP-G116C-6×His	15941.9	15942.0
FLAG-GABARAP-ACA	15272.7	15273.0
FLAG-GABARAPL2-G116C-6×His	15640.6	15640.7
FLAG-GABARAPL2-ACA	14970.8	14971.1
FLAG-MNSFβ(C57S)-G74C-6×His	9906.6	9906.9
FLAG-MNSFβ-ACA	9237.7	9237.9
